# Host-guest liquid gating mechanism with specific recognition interface behavior for universal quantitative chemical detection

**DOI:** 10.1038/s41467-022-29549-1

**Published:** 2022-04-07

**Authors:** Huimeng Wang, Yi Fan, Yaqi Hou, Baiyi Chen, Jinmei Lei, Shijie Yu, Xinyu Chen, Xu Hou

**Affiliations:** 1 State Key Laboratory of Physical Chemistry of Solid Surfaces, College of Chemistry and Chemical Engineering, Xiamen University, Xiamen, 361005 China; 2 Institute of Artificial Intelligence, Xiamen University, Xiamen, 361005 China; 3 Carbon Neutral Innovation Research Center, Xiamen University, Xiamen, 361005 China; 4 College of Physical Science and Technology, Xiamen University, Xiamen, 361005 China; 5 Innovation Laboratory for Sciences and Technologies of Energy Materials of Fujian Province (IKKEM), Xiamen, 361005 China

**Keywords:** Dynamic combinatorial chemistry, Sensors, Surface chemistry, Environmental monitoring

## Abstract

Universal visual quantitative chemical detection technology has emerged as an increasingly crucial tool for convenient testing with immediate results in the fields of environmental assessment, homeland security, clinical drug testing and health care, particularly in resource-limited settings. Here, we show a host-guest liquid gating mechanism to translate molecular interface recognition behavior into visually quantifiable detection signals. Quantitative chemical detection is achieved, which has obvious advantages for constructing a portable, affordable, on-site sensing platform to enable the visual quantitative testing of target molecules without optical/electrical equipment. Experiments and theoretical calculations confirm the specificity and scalability of the system. This mechanism can also be tailored by the rational design of host-guest complexes to quantitatively and visually detect various molecules. With the advantages of versatility and freedom from additional equipment, this detection mechanism has the potential to revolutionize environmental monitoring, food safety analysis, clinical drug testing, and more.

## Abstract (web summary)

In field, visual, chemical detection is of use for a wide range of possible applications. Here, the authors report on the creation of a host-guest liquid gating mechanism where detection of the target host triggers gate opening allowing for gas through the liquid gate, which can be used for visual detection.

## Introduction

Quantitative detection methods are useful to quantify and analyze chemical molecules in the environment and organisms, providing evaluation guidance regarding environmental assessment, food safety, health monitoring, clinical drug testing, and homeland security^1–4^. The principles of current analytical methods, such as the unique and innovative host-guest principle rely mainly on molecular recognition events, to convert information about the analytes into a quantifiable physical signal^5–7^. In the indicator displacement assay (IDAs) design, the use of the host-guest principle requires indicators that activate upon displacement from the receptor by a competitive analyte with a higher affinity, thereby resulting in the highly sensitive and specific conversion of molecular recognition signals to electrically detectable spectroscopic signals from indicators (for example, fluorescent, electrochemical, circular dichroism, or nuclear magnetic resonance (NMR) signals)^8–13^. The gas-liquid interface is the site of a wealth of chemical sensing signals, including chemical molecular recognition, reconstruction and assembly, and even changes in interfacial physical behavior, providing unprecedented opportunities for the development of sensing technology^14,15^. However, few analytical principles focus on the information at the gas-liquid interface, ignoring the physicochemical change parameters induced by interfacial molecular transformation mechanisms. A quantitative detection signal is also expected to be obtained from the information conversion of interface behavior, which will fully exploit the potential applications of relevant analytical principles, such as IDAs, to open the door for chemical detection. Analytical signal transduction without expensive optical detectors or electrochemical components facilitates the development of sensors for applications in resource-limited environments such as fieldwork or underdeveloped areas^16–18^. Therefore, it is significant for quantitative methods to construct a superior testing principle with intuitive and electricity-free molecular interface signal conversion and readout^19,20^.

The idea of using liquids as a structural and functional material to build responsive gates sounds counterintuitive, even verging on science fiction^21^. However, this idea has already become a reality. Liquid gating technology is an emerging technology that adopts a functional liquid as a structural material to build a reconfigurable gate with intrinsically differential response profiles to provide a unique combination of dynamic molecular reconfiguration and physicochemical interface behavior^22–24^. Recently, we have proposed a dipole-induced mechanism to explore the feasibility of ionic detection^25^. Although it achieves qualitative detection of different ions, this mechanism cannot achieve specific and quantitative detection by nature. This makes the strategy limited in real applications. Thus, it is a major challenge to seek a mechanism that can achieve the most important chemical detection requirements, including specificity and quantification.

**Fig. 1:**
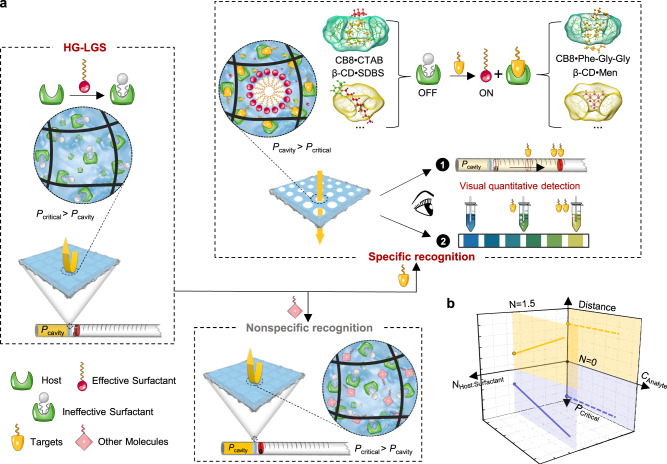
Host-guest liquid gating system (HG-LGS). **a** Schematic of the HG-LGS for quantitative visual chemical detection. The gating liquid consists of a host-guest system with a macrocyclic-surfactant. Macrocyclic molecules modulate the gas-liquid interface property of the gating liquid by shielding the surfactant to form an inclusion complex, in which the hydrophobic part of the surfactant is inserted into the macrocyclic cavity. When the specific target molecules are in the gating liquid, the HG-LGS releases gas with the formation of the macrocyclic-target complex, pushing the marker forward in the thin tube. The higher the concentration of the target molecule, the farther the marker moves. The concentration of target molecule can be visually quantified by reading the movement distance of the marker or observing the color change of indicator solution. The system does not respond to nonspecific molecules. **b** Relationships among the movement distance of the marker, the transmembrane critical pressure (*P*_Critical_) of the gas in the HG-LGS, and the concentration of the target molecule (*C*_Analyts_) in various macrocyclic: surfactant ratios (*N*_Host:Surfactant_).

Here, we present a host-guest liquid gating mechanism that can achieve quantitative detection with clear advantages, resulting in a portable, affordable on-site sensing platform for quantitatively testing target molecules without electrical equipment (Fig. 1a). This work successfully achieves not merely qualitative but quantitative chemical detection, which is one of the crucial achievements of liquid gating technology in chemical detection. Moreover, our experimental results and theoretical calculations have proven that the host-guest liquid gating system (HG-LGS) can selectively and quantitatively detect target molecules. This detection approach can also provide additional functionalities, such as the colorimetric vision detection of target molecules based on a color reaction from its quantitative gas release. Thus, we believe that such a versatile platform can also quantitatively detect a wide range of target molecules in various resource-limited settings, and has the potential to revolutionize environmental assessment, food safety analysis, clinical drug testing, and more.

## Results

### Mechanism of the HG-LGS

The behavior of a liquid gating system is based on reversible reconfigurable gates, which can use a capillary-driven functional gating liquid to seal microscale pores that can be opened at a certain pressure. For gas to pass through the liquid gating system, the pressure *P* must be greater than the gating threshold *P*_Critical_ (the smallest pressure needed to open the pores). Otherwise, the gas is prevented from passing through the system. Changes in the physical properties of functional gating liquids (e.g., surface tension) can be practically reflected by the transmembrane behavior of the gas. The HG-LGS is fabricated by impregnating the functional gating liquid that contains the host (macrocyclic molecules, such as cucurbituril and cyclodextrin) and guest (surfactants, such as hexadecyl trimethyl ammonium bromide and sodium dodecylbenzene sulfonate) in a hydrophilic nylon membrane. The selection of an appropriate host-guest system as the gating liquid in the HG-LGS is crucial. Macrocyclic molecules with high affinity and specific recognition will form specific host-guest couples with unique host-guest interactions. Surfactant molecules act as guest indicators because their surface activities are closely related to *P*_Critical_ in the HG-LGS. Surfactant molecules in solution usually reside preferentially at the interface with the interfacial structure of the hydrophobic end facing the air phase, allowing surface activity to reduce the system’s surface tension. The surface activity is shielded when the hydrophobic chain of the surfactant molecule enters the hydrophobic cavity of a macrocyclic molecule^26,27^, which provides a potential framework for the analyst-response mechanism. When a specific and competitive target molecule is present in the gating liquid, the surfactant indicator is displaced into the solution, where it occupies the surface, leading to a low *P*_Critical_. In contrast, nonspecific molecules (weak competitors) cannot displace the surfactant indicator, and the system still maintains a high *P*_Critical_ (Fig. 1a).

The HG-LGS can achieve visual, quantitative. Various *P*_Critical_ values, which reflect the transmembrane ability of the gas, have been quantified by the position change of a preloaded marker that advances by the released gas (air) in a thin tube. The concentrations of the target molecule can be utilized to control the amount of released gas (CO_2_), finally resulting in obvious changes in the color of the indicator solution. Figure 1b shows the relationships between the movement distance of the visual marker, the transmembrane critical pressure of gas in the HG-LGS, and the concentration of the target molecule at different macrocyclic: surfactant molar ratios. With no surfactant in the gating liquid (*N* = 0), the system shows no response to the target molecule. When the macrocyclic: surfactant ratio is 1.5, there is a linear relationship between the concentration of the target molecule and the movement distance of the marker. The mechanism of the HG-LGS ensures the direct conversion of target responses at the interface to obtain quantitative and visual parameters, which is quite different from the mechanisms of previously reported fluorescent or electrochemical sensors.

### Establishment of the HG-LGS

**Fig. 2:**
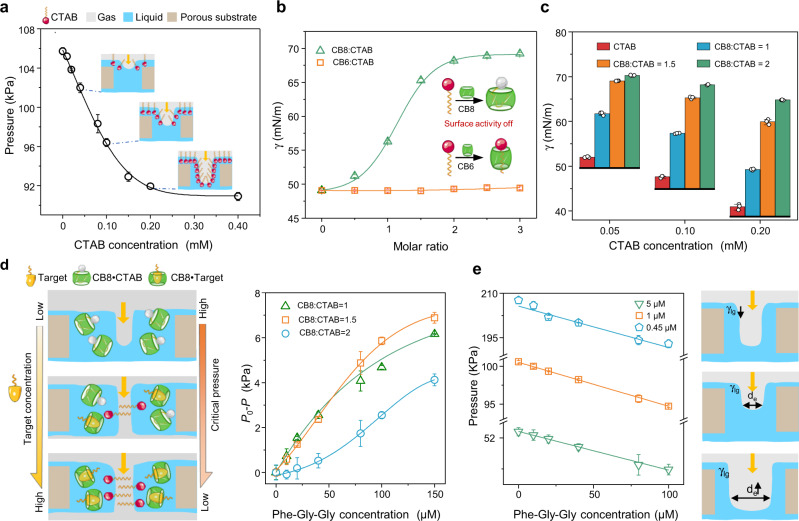
Establishment of the HG-LGS and its response to target molecules. **a** Variations in the critical pressure of the gas with CTAB concentration. **b** Ability of different cucurbiturils (CB8 and CB6) to shield the surface activity of the surfactant (CTAB = 0.1 mM). **c** Influence of the CB8: CTAB molar ratio on the surface tension at different CTAB concentrations. **d** Influence of the target molecules on the gating behavior of gas in the HG-LGS. Left: Illustration of the gating behavior of the target molecules. Different concentrations of Phe-Gly-Gly modulate the critical pressure of the gas. Right: Critical pressure of gas as a function of the Phe-Gly-Gly concentration at different CB8: CTAB ratios, where, *P* and *P*_0_ are the critical pressures of gas with and without Phe-Gly-Gly. **e** Effect of the pore size on the critical pressure of the HG-LGS for detection of Phe-Gly-Gly (CB8: CTAB = 1.5: 1, CTAB = 0.1 mM). Error bars represent standard deviations (*n* = 3).

Hexadecyl trimethyl ammonium Bromide (CTAB), an amphiphilic molecule that possesses a  long alkyl chain (hydrophobic end) and a quaternary ammonium group (hydrophilic end), is a key surfactant molecule in the establishment of the HG-LGS. When the CTAB solution is introduced into the gating liquid of the HG-LGS, increasing the CTAB concentration will reduce the surface tension of the gating liquid (Supplementary Fig. 1a). Stemming from the liquid gating mechanism, a decrease in the gas’s *P*_Critical_ then occurs (Fig. 2a). With different pore diameters, the *P*_Critical_ is also stably regulated by changing the CTAB concentration, verifying that CTAB reliably affects *P*_Critical_ and can serve as the unique molecule as the sensor indicator (Supplementary Fig. 2).

The host cavity is another important factor in establishing the HG-LGS, which determines the specificity and quantitative performance of sensors with host-guest interactions by adjusting the surface activity of the gating liquid. The surface tensions of CB[*n*] (*n* = 6 and 8):CTAB solutions were first measured to examine the surface activity of CTAB in the gating liquid by forming host-guest complexations (Fig. 2b). With increasing the CB8, the surface tension of the liquid gradually increases, and the largest change occurs at molar ratios ranging from 1:1 to 2:1. The surface tension is close to that of pure water when the molar ratio is 2:1. Upon the addition of more CB8, the surface tension no longer changes because the CTAB molecules are all in CB8 cavities, and no further CB8•CTAB complexation can occur. However, when CB6 is used, no change in the surface tension of the solution is observed regardless of the CB6:CTAB molar ratio. The reasons for the differences are as follows: (i) the alkyl chain of CTAB can insert into the CB8 cavity by adopting a U-shaped conformation, which shields the hydrophobic part of CTAB to establish a CTAB-deficient interface; (ii) the alkyl chain of CTAB has a straight-chain structure in the cavity of CB6, which forms an unstable CB6•CTAB structure that results in a weak van der Waals contact between the alkyl chain and inner wall of CB6^26^. The above conjecture is also verified by the optimized binding geometry of the CB8•CTAB complex and CB6•CTAB complex (Supplementary Fig. 3). The results show that the hydrophobic chains of CTAB are more likely to undergo conformational changes in the presence of CB8 and eventually change the gas-liquid interface behavior of the gating liquid. A Job plot analysis confirms a 1:1 stoichiometry of the complex formed between CB8 and CTAB (Supplementary Fig. 4). Choosing the proper CTAB concentration for a certain molar ratio of CB8:CTAB is crucial, affecting the sensitivity and range of detection. Figure 2c shows that at different CTAB concentrations, with an increasing CB8: CTAB molar ratio, all the surface tensions display the same increases, and the maximum ∆γ values of CTAB solutions with concentrations of 0.05 mM, 0.10 mM, and 0.20 mM are 18.27 mN/m (CB8: CTAB = 1), 20.55 mN/m (CB8: CTAB = 1.5) and 23.86 mN/m (CB8: CTAB = 2), respectively. The detection sensitivity is higher when the CTAB concentration is 0.1 mM or 0.2 mM. Because the critical pressure of gas decreases linearly from 0 to 0.1 mM (Supplementary Fig. 1b), 0.1 mM CTAB was selected.

### Target molecule influence on the gating behavior of the HG-LGS

Cucurbiturils have been reported to bind many biologically, medically, and environmentally relevant analytes^28–31^. A target molecule should have a high binding affinity with cucurbituril and the ability to free the surfactant molecule from the formed cucurbituril•surfactant in the host-guest interaction. As a proof-of-concept study, we chose phenylalanine-glycine-glycine (Phe-Gly-Gly) as the molecular recognition model to test the feasibility of the HG-LGS. The Phe-Gly-Gly molecule is a well-known guest of CB8 and is usually detected by IDAs and NMR^13,32^. The critical pressure of the gas and the surface tension of the gating liquid in the HG-LGS both gradually decrease with increasing Phe-Gly-Gly concentration (Supplementary Fig. 5a). This result reveals that Phe-Gly-Gly can effectively affect the gating behavior of HG-LGS by displacing the surfactant in the host-guest interaction (on the left of Fig. 2d). The right panel in Fig. 2d shows the target response during the critical pressure change at different CB8: CTAB molar ratios. It is worth mentioning that the *P*_0_*-P* value is different at different CB8: CTAB molar ratios. At 2:1, the *P*_0_*-P* value exhibits a slowly increasing trend, mainly stemming from the high quantity of free CB8 at the beginning. At a ratio of 1.5:1, the pressure value and analyte concentration exhibit a linear relationship in the concentration range of 0–100 μM (Supplementary Fig. 5b). There is a 588 Pa pressure drop when the concentration of Phe-Gly-Gly decreases to 10 μM, which is sufficient for sensing the target molecule.

In addition, the competitive binding between CB8•CTAB and guests with different binding affinities is further investigated. The results show that those potential interfering molecules (metal ion, cholesterol, and fatty acid) do not affect the gating behavior of the system. While these guests with a high binding affinity to CB8 can displace the surfactant to change the gating behavior and can be used as target molecules (Supplementary Fig. 6). Such as, memantine, a drug molecule for the treatment of Alzheimer’s disease, can effectively affect the gating behavior in the HG-LGS by replacing the surfactant (Supplementary Fig. 7).

The pore size of porous membranes is vital in determining the detection sensitivity. There is always a linear relationship between the critical pressure and the analyte concentration with different pore diameters (Fig. 2e). The smaller the membrane pore size is, the higher the critical pressure and the more sensitive the analyte response.

### Competitive binding mechanisms with CB8•CTAB

**Fig. 3:**
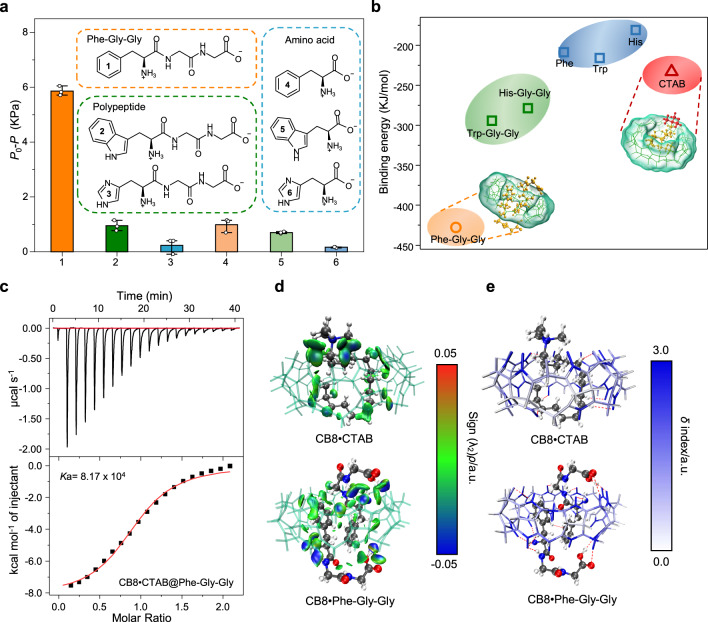
Competitive binding mechanisms with CB8•CTAB. **a** Selectivity of the HG-LGS with various molecules: (1) Phe-Gly-Gly; (2) Trp-Gly-Gly; (3) His-Gly-Gly; (4) Phe; (5) Trp; and (6) His. **b** Plots of the binding energy between CB8 and various guest molecules. **c** ITC data for CB8**•**CTAB complexation with Phe-Gly-Gly at 25 °C in 10 mM phosphate buffer, pH = 7.0. **d** *δ*g^inter^ = 0.01 a.u. isosurfaces colored by the sign of (λ_2_)*ρ* for the CB8•CTAB and CB8•Phe-Gly-Gly complexes. Blue represents strong attraction, green represents van der Waals forces, and red represents strong repulsion. **e** Atoms of CB8 colored based on their contribution to binding with CTAB and Phe-Gly-Gly. Gray represents no contribution to the complexation, while blue represents the largest relative contribution. Error bars represent standard deviations (*n* = 3).

Urbach and coworkers have explored a series of molecular recognition systems using sequence-specific peptides (e.g., N-terminal tryptophan, N-terminal aromatic peptides, tripeptides) with CB8 by testing the binding affinities and have discussed the selective recognition mechanisms^11,13,33,34^. However, which molecule is preferred in competitive binding with CB8•CTAB in the presence of a surfactant guest needs further confirmation. The interactions of various molecules with hydrophilic or hydrophobic ends with CB8•CTAB in the gating liquid were observed. As shown in Fig. 3a, compared with the target molecules, no obvious changes in the critical pressure of gas are observed with the interfering molecules (Trp-Gly-Gly, His-Gly-Gly, Phe, Trp and His). The surface tension results are also consistent (Supplementary Fig. 8).

We further compared the binding energies of different complexes from thermodynamics to quantitatively evaluate the competitive effects of various guest molecules with CB8. Semiempirical quantum mechanical methods using the Molclus program with all-electron density functional theory were used to search for the optimal complex structures^35^. Implicit solvation models based on density (SMD) were used to describe the effect of the aqueous solution environment on the host-guest molecular configurations^36^. The SMD includes a polar part describing the bulk electrostatic interaction and a nonpolar part describing solvent cavitation and possible changes in the local solvent structure, which can virtually describe the structural changes of water molecules around the complex molecules^37^. Here, we chose the calculation method (B3LYP with D3BJ dispersion) with suitable basis sets (6-311 G (d, p)) to calculate and obtain the relatively accurate binding energy value. Although these computed binding energies are not precise in absolute value, they can provide the right trend of our different systems. The binding energies were plotted in Fig. 3b. The results show that CB8 is more likely to bind to two Phe-Gly-Gly molecules, and has the lowest binding energy (−427 kJ mol^−1^) compared to other tripeptides (Trp-Gly-Gly and His-Gly-Gly), amino acids (Phe, Trp,and His), and surfactant CTAB (−232 kJ mol^−1^). The lower the binding energy is, the better the thermodynamic stability of the complex, and the more likely it exists in the system. This means that Phe-Gly-Gly can drive the CTAB indicator to leave the cavity of CB8, inducing a change in the transmembrane behavior of the gas.

The binding thermodynamics of CB8•CTAB to various amino acids and peptides were also evaluated by isothermal titration calorimetry (ITC). Unexpectedly, only the binding constant of Phe-Gly-Gly was obtained. The equilibrium association constants for other molecules were <10^3 ^M^−1^ (Fig. 3c and Supplementary Fig. 9). These results are inconsistent with the previously reported recognition of amino acids and peptides by the CB8 or CB8•MV complex^34^. This is probably due to the presence of CTAB, which affects the binding affinity of CB8 for guests.

An independent gradient model (IGM)^38^ that relies on Multiwfn software^39^ was used to investigate the specific molecular interactions of CB8•CTAB and CB8•Phe-Gly-Gly. This model reveals that CTAB is in the U-shaped conformation in CB8•CTAB, having the maximum van der Waals force between the alkyl chain and CB8 cavity and simultaneously forming more hydrogen bonds between the alkyl chain and carbonyl oxygen of CB8 (Fig. 3d). Regarding CB8•Phe-Gly-Gly, two peptides enter the cavity from opposite openings, and the phenyl groups contact CB8 via van der Waals interactions. Two phenyl groups have a positive synergistic effect on the formation of the complex. N-H…O hydrogen bonds and van der Waals interactions are observed between the Gly-Gly tails and CB8, indicating that a long peptide tail may improve the binding affinity between CB8 and peptides. This IGM model result is consistent with the previously reported selective recognition of Phe-Gly-Gly for CB8 as revealed by the crystal structure^13^. Further coloring of the atoms involved in CB8 that join in the host-guest complexation clearly shows that the major interaction sites derive from the carbonyl group (Fig. 3e).

### Quantitative visual detection of Phe-Gly-Gly

**Fig. 4:**
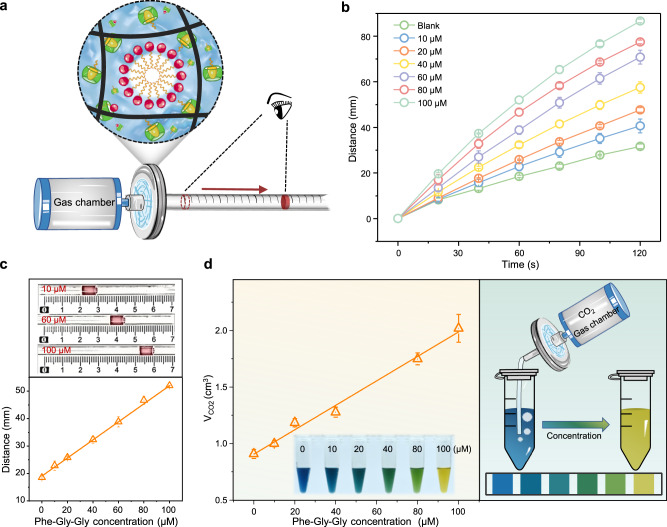
Performance of HG-LGS in Phe-Gly-Gly detection and quantitative CO_2_ release. **a** Schematic for quantifiable visual detection based on the distance signal readout. **b** Time-dependent marker advancement with different concentrations of Phe-Gly-Gly. **c** Linear standard curves obtained from 0 to 100 μM Phe-Gly-Gly in 1 min. Inserts: Images showing marker advancement at various concentrations of Phe-Gly-Gly in 1 min. **d** Quantifiable visual CO_2_ release based on the color signal readout. Left: Plots of CO_2_ emission against the Phe-Gly-Gly concentration. Right: Illustration of the color signal readout by the HG-LGS. Error bars represent standard deviations (*n* = 3).

A simple device was designed to realize the transformation of host-guest molecular recognition into quantitative and visual parameters. The marker is propelled by gas (air) released from the chamber to indicate the critical pressure change caused by the variation in the concentration of the target molecule in HG-LGS, enabling visual detection (Fig. 4a). The time dependence of the marker movement was plotted in Fig. 4b, and the result indicates that the movement rate of the marker is positively correlated with the Phe-Gly-Gly concentration. A high Phe-Gly-Gly concentration causes more displacement of the surfactant molecules and a clear decrease in the critical pressure of the HG-LGS sensor, resulting in rapid movement of the marker. A longer distance of movement reflects a lower critical pressure in the system and a higher target analyte concentration. With 100 μM Phe-Gly-Gly, the marker can move 52.0 mm in one minute. With the 10 μM Phe-Gly-Gly, the marker moves only 22.8 mm in one minute (Fig. 4c). The HG-LGS sensor has an excellent quantitative detection ability with a linear range of 0–100 μM. The limit of detection (LOD) is 4.56 μM, according to the 3σ/slope rule (*N* = 3). With a 5 μm membrane, the LOD of the HG-LGS sensor is 7.98 μM (Supplementary Fig. 10). We also find a linear relationship between the change in chamber pressure and the target molecule concentration, which can be measured by a portable pressure gauge (Supplementary Fig. 11). These results prove that this system can successfully transform the molecular recognition signal into a quantitative and visual physical signal by combining host-guest interactions and liquid gating technology.

### Quantitative substance release by the HG-LGS

Drug delivery systems with stimuli-responsive release abilities are highly desired in the pharmaceutical and biomedical fields. As a proof-of-concept, a device based on the HG-LGS was designed to verify the feasibility of regular substance release. The successful quantitative release of carbon dioxide (CO_2_) depends on the control of the target molecule in the gating liquid to change the transmembrane behavior (Fig. 4d). CO_2_ is the transport substance, and bromothymol blue solution is the carbon dioxide indicator. A calibration curve is established between the CO_2_ flux and target molecule concentration. Increasing the concentration of the target, increases the CO_2_ flux, and the color of the carbon dioxide indicator changes from blue to green to yellow. The concentration values of target molecules can be expressed by different colors, which are easily recognizable with the naked eye. The HG-LGS has the potential to control drug release by quantitatively regulating the target concentration to obtain visual color results. Integration into artificial intelligence wearable devices that can implement the stimulus-response release mechanism of the HG-LGS will provide a potential method for drug detection and treatment^40,41^.

### Specific detection of memantine with the HG-LGS

**Fig. 5:**
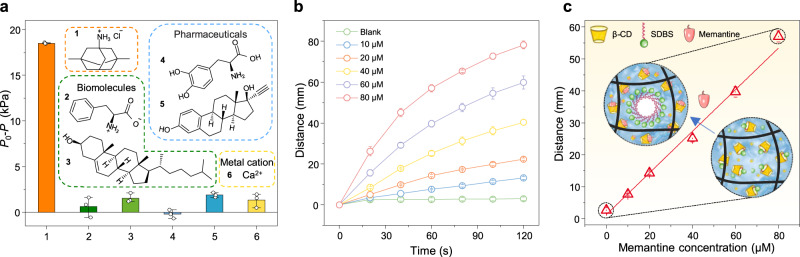
Performance of the HG-LGS for memantine detection. **a** Selectivity of memantine detection by the HG-LGS**:** (1) memantine; (2) amino acids; (3) cholesterol; (4) levodopa; (5) ethinylestradiol; and (6) Ca^2+^. **b** Time-dependent marker movement with different concentrations of memantine. **c** Linear standard curves obtained from 0–80 μM memantine within 1 min. Inserts: Schematic diagram of memantine detection by the HG-LGS with β-CD•SDBS and β-CD•memantine host-guest interactions. Error bars represent standard deviations (*n* = 3).

To explore the generality and versatility of the HG-LGS, we used β-cyclodextrin (β-CD)-sodium dodecylbenzene sulfonate (SDBS) as a host-guest system to detect memantine. β-CD, similar to cucurbit[8]uril, has a hydrophobic cavity and can combine with SDBS to regulate the surface activity of liquids. The surface tension of the system reaches the maximum value when the molar ratio of β-CD: SDBS is 2:1, where the surface tension is close to that of pure water (Supplementary Fig. 12). According to the binding energy from the simulation results, memantine binds β-CD with a stronger affinity than that of the indicator molecule SDBS, which was used as the analyte (Supplementary Fig. 13). When memantine is present, SDBS is displaced by the formation of β-CD•memantine, resulting in a decrease in the critical pressure (Supplementary Fig. 14). To evaluate the selectivity of the designed HG-LGS for memantine detection, we also explored the response of the HG-LGS to some potential interferents (Fig. 5a). The sensor exhibited selectivity to memantine over other interfering molecules, such as biomolecules (amino acids and cholesterol), pharmaceuticals (levodopa and ethinylestradiol), and metal cations (Ca^2+^). Moreover, experiments were conducted to detect memantine in urine with the HG-LGS to prove the feasibility of target detection in complex biofluids. Urine samples containing a series of concentrations of memantine were tested by the HG-LGS. The target molecule memantine still effectively displaced the surfactant from the host cavity to change the system’s surface tension and critical transmembrane pressure of the gas (Supplementary Fig. 15). These results ensure that the HG-LGS can operate in complex biofluidic environments, which is attractive for practical applications. Various concentrations of memantine in the gating liquid can be successfully detected based on the movement distance of the marker. As shown in Fig. 5b, the movement rate of the marker gradually increases with increasing amounts of memantine. The movement distance of the marker has a linear relation to the memantine concentration, which enables the quantitative detection of memantine with the HG-LGS (Fig. 5c). The detection limit is 3.52 μM, and the detection range is 0–80 μM. The detection mechanism developed here can visually quantify many other targets because functionalized macrocyclic molecules can bind a broad range of targets^42,43^.

## Discussion

In summary, we have demonstrated a specific recognition interface behavior mechanism that converts molecular interface recognition behavior to visually quantifiable detection signals. The detection mechanism inherently addresses key challenges in terms of universality, portability, and visibility while requiring no energy input for target molecule detection. Unlike traditional detection based on fluorescence molecules as an indicator, the HG-LGS utilizes a dynamic gas-liquid interface to convert host-guest interactions into gas transmembrane behavior, which is expressed by visual and quantitative physical parameters. The approach of stimulating an interfacial change in a gating liquid can also be used for colorimetric detection or drug release control. Moreover, the HG-LGS is validated as a highly scalable system that can be customized to detect a broad range of target molecules because it can integrate with various host-guest molecule bindings. We envision that this mechanism will open avenues for more in-depth exploration of chemical detection and spur advances in environmental monitoring, point-of-care test, public health security, and biomedical applications.

## Methods

### Chemicals

Cucurbit[8]uril (CB8), cucurbit[6]uril (CB6), β-Cyclodextrin (β-CD), hexadecyl trimethyl ammonium bromide (CTAB), L-phenylalanine (Phe), L-tryptophan (Trp), and L-histidine (His) were purchased from Macklin. Sodium dodecylbenzene sulfonate (SDBS) was purchased from Sinopharm Chemical Reagent Co., Ltd. H-Phe -Gly-Gly-OH was purchased from Shanghai Fusheng Industrial Co., Ltd. H-Trp -Gly-Gly-OH and H-His -Gly-Gly-OH were purchased from GL Biochem (Shanghai) Ltd. Memantine hydrochloride was purchased from Dalian Meilun Biotech Co., Ltd. Bromothymol blue was purchased from Beijing Solaibao Technology Co., Ltd. Deuterium oxide was purchased from J&K Scientific. Cholesterol was purchased from Sangon Biotech (Shanghai) Co., Ltd. Levodopa, and Ethynylestradiol was purchased from Beijing Xinhengyan Technology Co., Ltd. Nandrolone was purchased from Abmole Bioscience Inc. Propranolol Hydrochloride was purchased from Beijing Huawei Ruike Chemical Co., LTD Ltd. The above commercial reagents of analytical grade were used without further purification. Urine samples were carefully taken from healthy individuals and stored at 4 °C. A variety of hydrophilic organic nylon 66 membranes were purchased from Haining Zhongli Filtering Equipment Factory. Mili-Q deionized (DI) water was obtained from Mili-Q Integral 3 System (18.2 MΩ cm). A 100 mM phosphate buffer stock solution was adjusted to pH 7.0 and sterile. All CB8•CTAB experiments described here were performed in 10 mM phosphate buffer, prepared by diluting 100 mM.

### Fabrication of liquid gating membranes

The CB8•CTAB functional liquid was prepared by mixing the two compounds CB8•CTAB (1.5: 1, CTAB 1 mM) in 10 mM phosphate buffer solution (pH = 7.0). After 10 min of ultrasonic treatment, the solution was incubated at room temperature for 24 h to form a stable complex. The β-CD•SDBS functional liquid was prepared by mixing the two compounds β-CD•SDBS (1.5: 1, SDBS 0.2 mM) in deionized water. After ultrasonic treatment for 10 min and incubation at room temperature for 24 h, stable β-CD•SDBS complexes were formed. Finally, the liquid gating membranes were generated by infusing the functional liquid into various hydrophilic organic nylon 66 membranes (0.45, 1, and 5 μm).

### Surface tension measurements

The surface tension was directly measured via the pendant drop method on the OCA100 system at 25 ^o^C. The surface tension data were recorded with droplet volume as large as possible. The same sample was repeated at least three times in all experiments.

### Transmembrane critical pressure measurements

The gating properties of the HG-LGS were determined by measuring the transmembrane critical pressure during the flow of gas with a syringe pump (Harvard Apparatus PHD ULTRA). Unless specified otherwise, all gas transmembrane experiments were performed using air. The pressure was measured by wet/wet current output pressure transmitters (PX273-100DI) from OMEGA Engineering Inc. (Stamford, CT, USA). During testing, the syringe pump with a flow rate of 2000 μL min^−1^, and an organic nylon 66 membrane in a circular shape of 25 mm in diameter was used in all experiments. Unless otherwise specified, the pore size of organic nylon 66 membranes used in HG-LGS is 1 μm. For each system, the same sample was tested for pressure data at least three times under the same conditions.

### Isothermal titration calorimetry (ITC)

All ITC experiments were carried out using MicroCal ITC 200 microcalorimeter (GE15 Healthcare) in 10 mM phosphate buffer (pH 7.0, 25 °C). In a typical experiment, the CB8•CTAB (0.15 mM: 0.1 mM) was placed in a reaction cell, and each analyte (2 mM) was injected 19 times (2 µL per time) into CB8•CTAB solutions with stirring. In the CB8 binding CTAB experiment, the CB8 (0.1 mM) was in the reaction cell, and CTAB (2.5 mM) was injected 19 times (2 µL per time) into CB8 solutions with stirring. The data were analyzed by Origin software.

### Visual quantitative detection

The HG-LGS sensor was prepared by assembling a gas chamber, silicone tubes, and filter membrane support device. The nylon membrane was immersed in the analytical sample solution, then sealed in the filter membrane support device. A certain amount of gas (air) was stored in the gas chamber by the syringe pump, and the pressure in the chamber was *P*_A_. A pressure sensor was used to measure pressure in real-time, and a camera was used to record the movement of the marker. For controlled release experiments, change the air in the gas chamber to CO_2_, and monitor the amount of CO_2_ released with a bromothymol blue indicator.

### Quantum chemical calculations

To find out the optimal structures of each complex, the configuration search was performed using the Molclus program. For each peptide and CB8, more than 2000 original structures were generated and optimized based on the semiempirical quantum mechanical methods GFN1-xTB^44^ and GFN2-xTB^45^. Then the ten lowest energy configurations were further optimized by an all-electron density functional theory (DFT) study using the hybrid density functional B3LYP together with the basic set of 6-31 G(d). Then, the single point energies and binding energies based on the structure with the lowest energy were further calculated under B3LYP/6-311 G (d, p) level. The all-electron DFT calculations were carried out using Gaussian 16 program^46^. To describe the weak interactions more accurately, all the calculations were with Grimme’s D3BJ dispersion. Here we took the solvent effects of water (ε = 78.3553) into account by using the SMD-flavor of self-consistent reaction field (SCRF) theory. The binding natures in the studied complexes were revealed with independent gradient model (IGM)^38^ using the software Multiwfn^39^, and binding energies were obtained based on the following equation:

$${E}_{{{{{{\rm{Binding}}}}}}}={E}_{{{{{{\rm{Complex}}}}}}}-\left({E}_{{{{{{\rm{CB}}}}}}8}+{E}_{{{{{{\rm{Peptide}}}}}}}\right)$$

All the visualization of molecular structures and contour surfaces were performed by VMD^47^.

### Reporting summary

Further information on research design is available in the Nature Research Reporting Summary linked to this article.

## Supplementary information

Supplementary Information

Reporting Summary

## Data Availability

Data within the manuscript and its Supplementary Information are available from the corresponding author upon reasonable request. The source data underlying Figs. 2a–e, 3a, b, 4b–d and 5a–c and Supplementary Figs. 1, 2, 4–8 and 10–15 are provided as a Source Data file. Source data are provided with this paper.

## Source data

Source Data
