# Supplementary material for: Host-guest liquid gating mechanism with specific recognition interface behavior for universal quantitative chemical detection
Source: Nat Commun. 2022 Apr 7;13:1906. doi: 10.1038/s41467-022-29549-1 (PMC8991241; doi:10.1038/s41467-022-29549-1)
Supplement: Supplementary file 1 — Supplementary Information [file 41467_2022_29549_MOESM1_ESM.pdf]

SUPPLEMENTARY INFORMATION

Host-Guest Liquid Gating Mechanism with Specific Recognition Interface Behavior for Universal Quantitative Chemical Detection

*Huimeng Wang[1], Yi Fan[1], Yaqi Hou[1,2], Baiyi Chen[1], Jinmei Lei[1], Shijie, Yu[1], Xinyu Chen[3] and Xu Hou[1,2,4,5]**

[1]State Key Laboratory of Physical Chemistry of Solid Surfaces, College of Chemistry and Chemical Engineering, Xiamen University, Xiamen 361005, China

[2]Institute of Artificial Intelligence, Xiamen University, Xiamen 361005, China

[3]Carbon Neutral Innovation Research Center, Xiamen University, Xiamen, 361005, China

[4]College of Physical Science and Technology, Xiamen University, Xiamen 361005, China

[5]Innovation Laboratory for Sciences and Technologies of Energy Materials of Fujian Province (IKKEM), Xiamen 361005, China

*Corresponding author. houx@xmu.edu.cn

SUPPLEMENTARY METHODS

Solvation model based on density (SMD)

We used one of the implicit solvation models, the solvation model based on density (SMD), to describe the effect of the aqueous solution environment on the host-guest molecular configurations. The SMD consists of two main components; one is the bulk electrostatic contribution ΔG_{ENP}, which is the same as that in the previous IEFPCM model; the other is the cavity-dispersion-solvent-structure term ΔG_{CDS}, which contains the entropic effects and is related to the atomic surface tension σ_A and solvent-accessible surface area S_A:

$$\Delta G_{CDS} = \sum_{A}^{atoms} S_A \sigma_A$$

in which the parameter σ_A is obtained by fitting experimental results of the solvation free energy. The SMD model is one of the implicit solvent models with the best accuracy, so it is selected to describe the effect of the water environment in this work.

^{1}H NMR Spectroscopy:

All spectra were collected in deuterium oxide at 25 °C on a Bruker AVANCE NEO 500 MHz spectrophotometer. In order to determine the stoichiometry of the CB8•CTAB complex by Jobs Plot, 11 samples with the mole fraction varying from 0 to 1.0 were prepared. At the same time, the total concentration of CB8: CTAB was maintained at 0.13 mM for each sample.

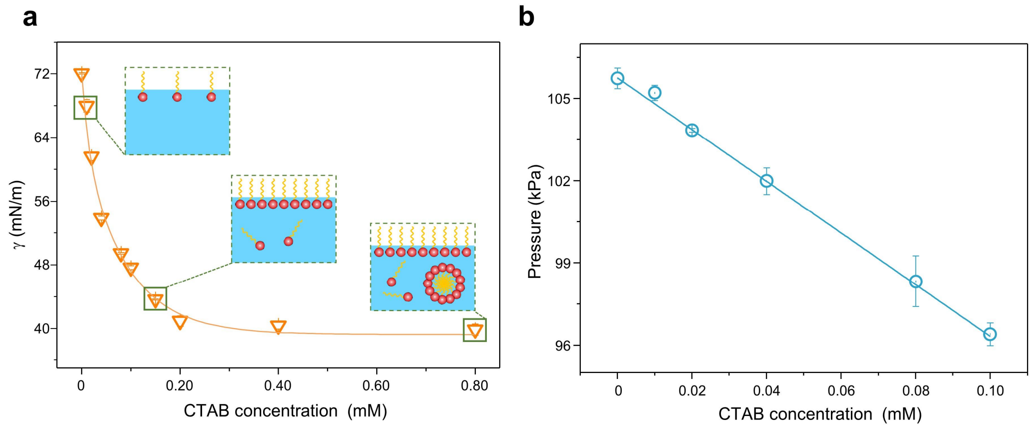

Supplementary Figure 1. a Surface tensions of the CTAB solution (0-0.8 mM). When the concentration increases to the critical micelle concentration, the surface tension of the solution sharply decreases from 72.0 mN/m to 40.9 mN/m. **b** Critical pressure of gas variance with the CTAB concentration. The Critical pressure of the CTAB shows a linear trend in the concentration range of 0-0.1 mM. Error bars represent standard deviations (n=3).

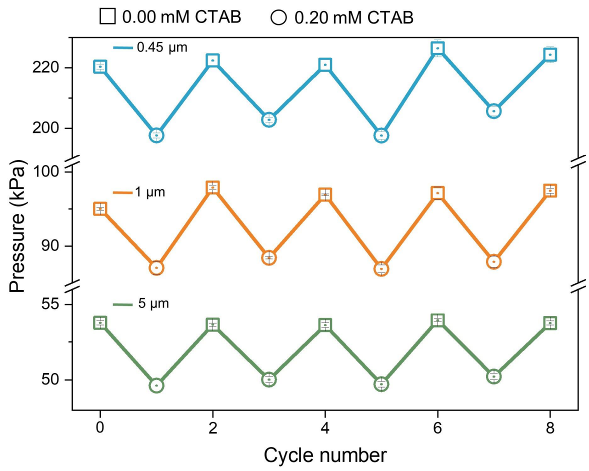

Supplementary Figure 2. Stability test of the HG-LGS without (circle) and with CTAB (0.2 mM CTAB solution, square) in nylon membranes with various pore sizes (0.45, 1, and 5 μm). Under different pore sizes, the critical pressure changed significantly with the CTAB concentration, demonstrating a stable and tunable gating response. Error bars represent standard deviations (n=3).

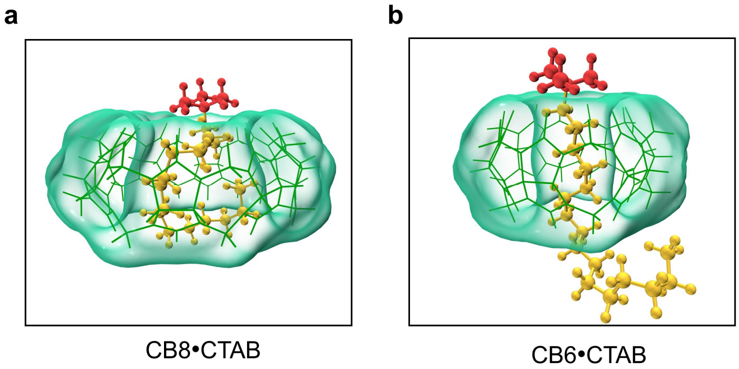

Supplementary Figure 3. The optimized binding geometry of CB8-CTAB complex and CB6-CTAB complex. **a** The hydrophobic end of CTAB is curled up in the CB8 cavity in a U-shaped structure, and its surface activity is shielded. **b** The hydrophobic end of CTAB is exposed outside the CB6 cavity, which is in contact with the solution and retains surface activity.

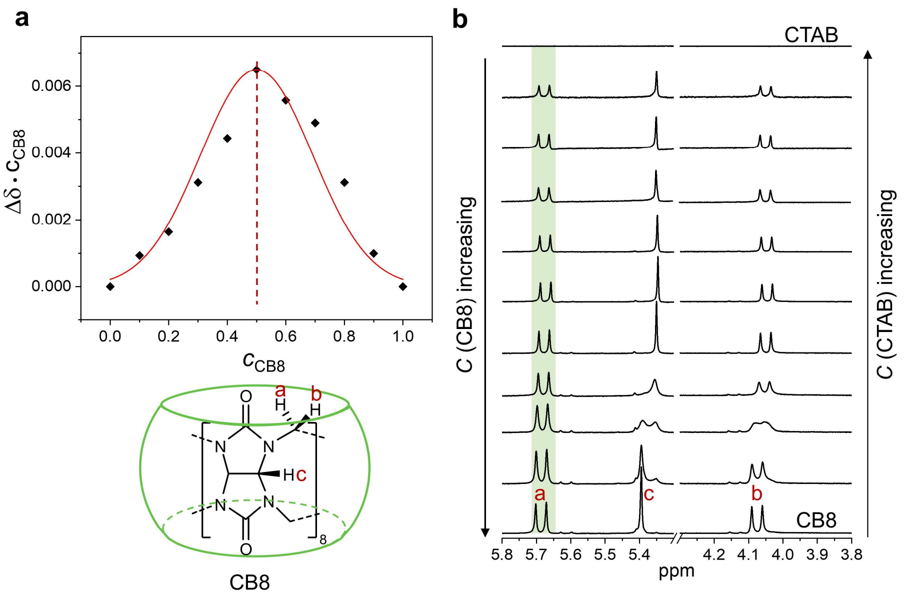

Supplementary Figure 4. NMR studies for CB8•CTAB complexation. **a** NMR Job plot for the CB8•CTAB complexation based on the shift of the H-a signal of CB8, indicating a 1:1 binary complexation. **b** Stacked NMR spectra for the complex formation of CB8•CTAB at different mole fractions.

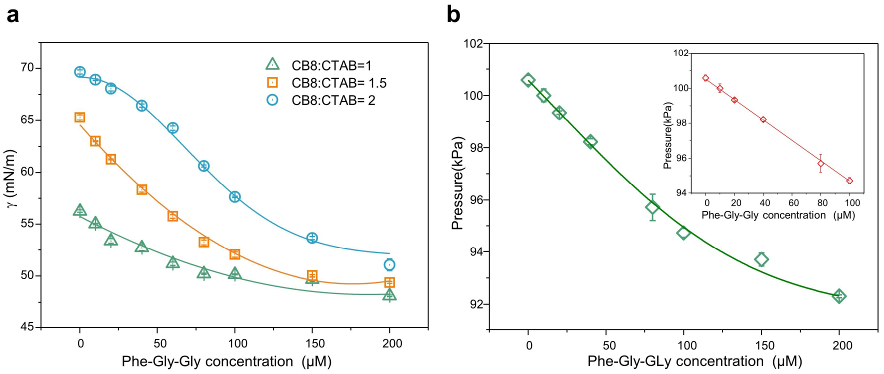

Supplementary Figure 5. a Surface tensions as a function of the Phe-Gly-Gly concentration with different molar ratios of CB8 to CTAB. **b** Critical pressure variance with the Phe-Gly-Gly concentration (molar ratio of CB8 to CTAB is 1.5). Inset: The linear relationship between the critical pressure and the Phe-Gly-Gly concentration (0-100 μM). The target molecule Phe-Gly-Gly that binds more strongly with CB8 displaced the surfactant CTAB from the CB8 cavity into the solution, which changes the interface properties of the HG-LGS system. Error bars represent standard deviations (n=3).

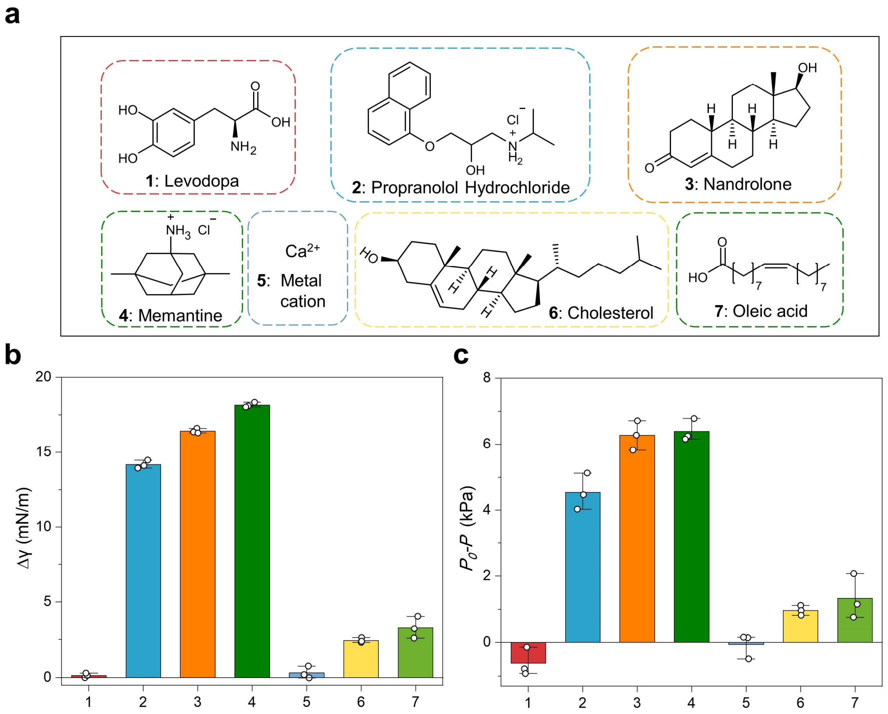

Supplementary Figure 6. a Guests for competitive interactions with CB8•CTAB in the HG-LGS. **b** Surface tension variation with different guest molecules. **c** Critical pressure variation with different guest molecules. Error bars represent standard deviations (n=3).

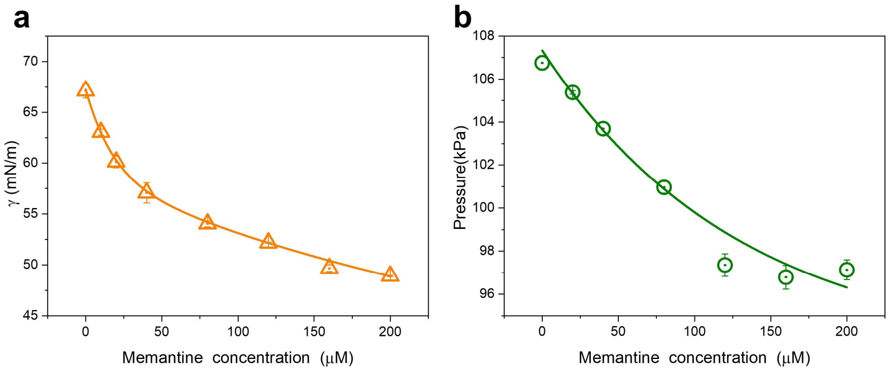

Supplementary Figure7. a Surface tensions of system variance with memantine concentration. **b** Critical pressure of system variance with memantine concentration (0-200 μM). Memantine can displace the CTAB in the cavity of CB8, resulting in the decrease of critical pressure of the system. Error bars represent standard deviations (n=3).

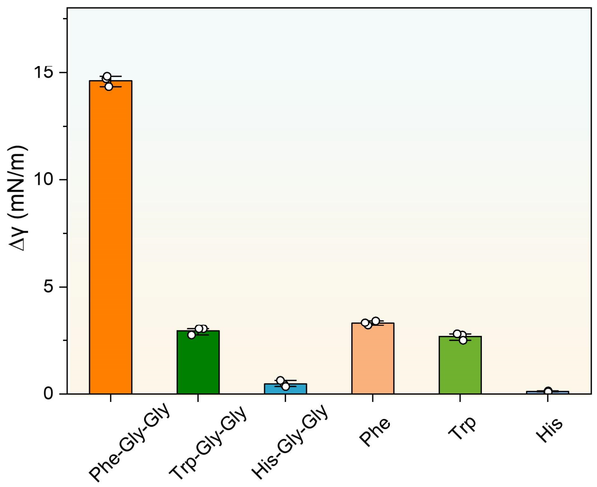

Supplementary Figure 8. Surface tensions change of CB8•CTAB after adding Phe-Gly-Gly and interfering molecules (Trp-Gly-Gly, His-Gly-Gly, Phe, Trp and His). Compared with other interfering molecules, the HG-LGS system exhibited better selectivity to Phe-Gly-Gly. Error bars represent standard deviations (n=3).

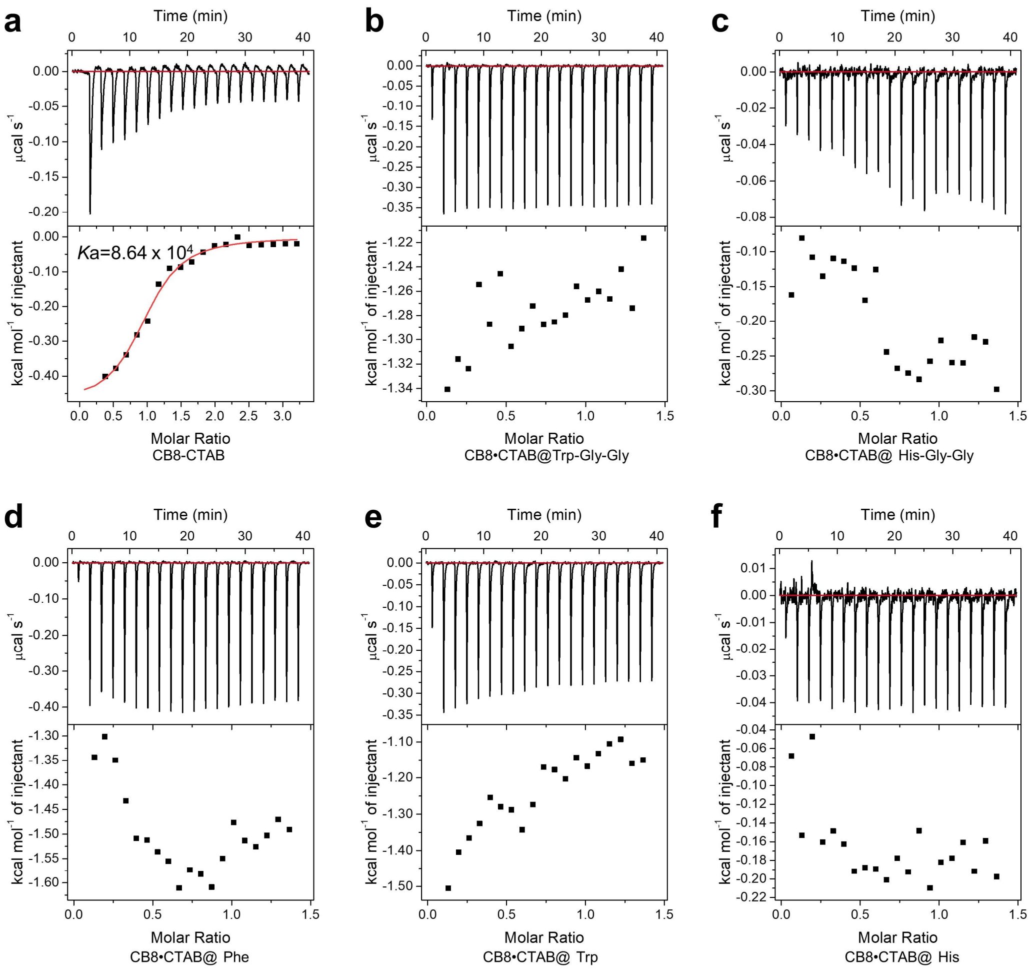

Supplementary Figure 9. ITC data of various complexes. **a** CTAB binding experiment was combined with CB8. **b-f** Trp-Gly-Gly, His-Gly-Gly, Phe, Trp, His binding experiments were combined with CB8-CTAB, respectively. The results showed no binding, indicating that the binding affinity was <10^3 M^{-1}.

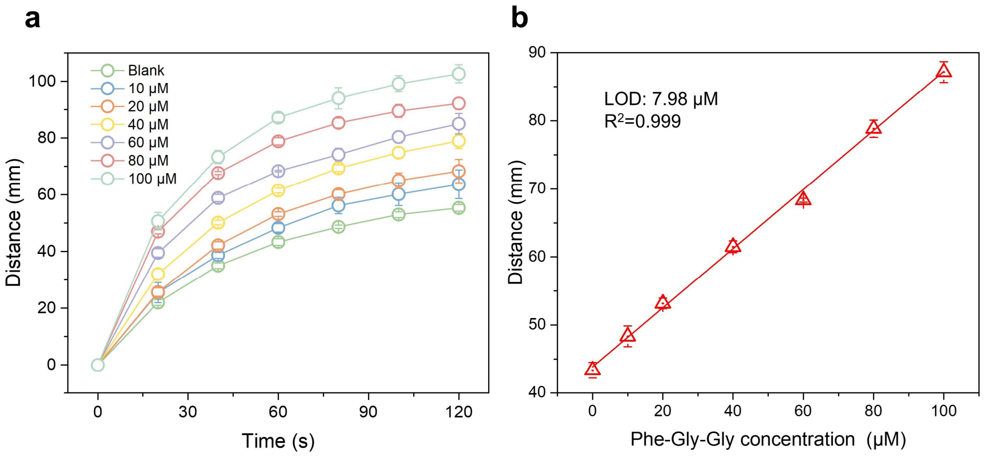

Supplementary Figure 10. a Time-dependent marker advancement with different concentrations of Phe-Gly-Gly. **b** Linear standard curves were obtained from 0 to 100 μM Phe-Gly-Gly in 1 min. pore size: 5 μm. Error bars represent standard deviations (n=3).

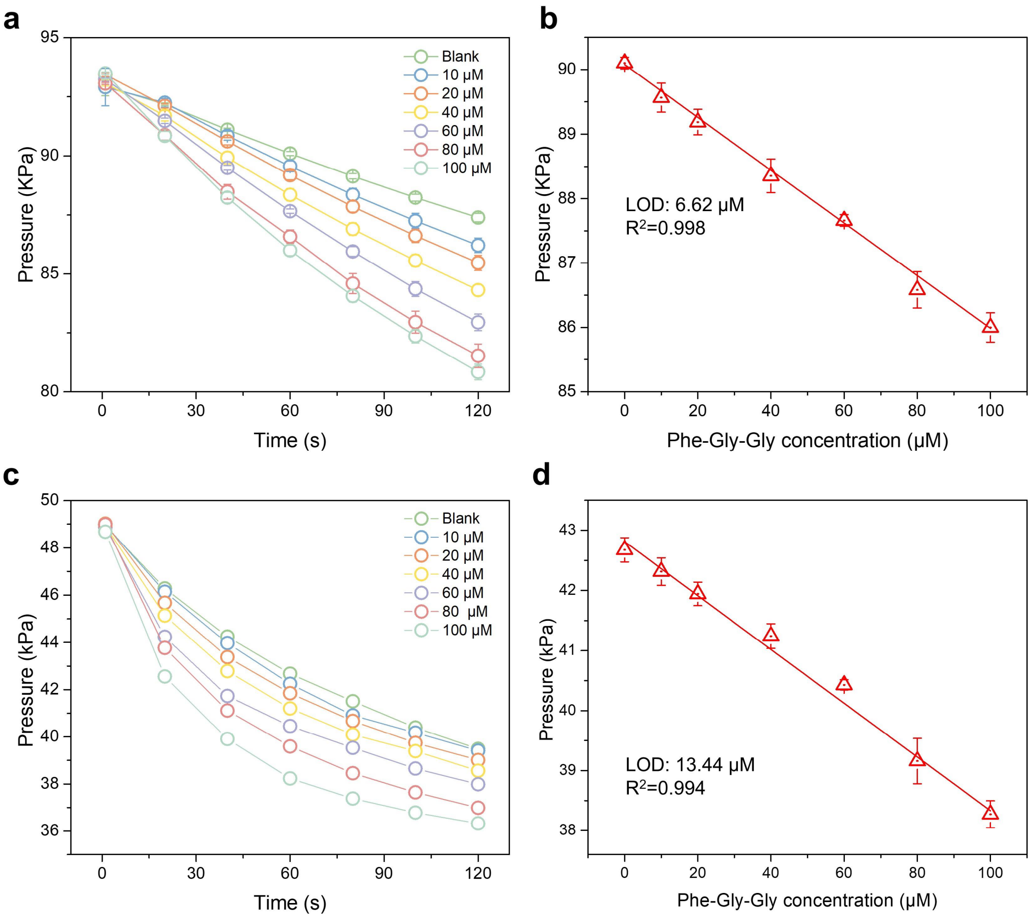

Supplementary Figure 11. a (pore size: 1 μm), **c** (pore size: 5 μm) Time-dependent pressure changes with different concentrations of Phe-Gly-Gly. **b** (pore size: 1 μm), **d** (pore size: 5 μm) Linear standard curves were obtained from 0 to 100 μM Phe-Gly-Gly in 1 min. Error bars represent standard deviations (n=3).

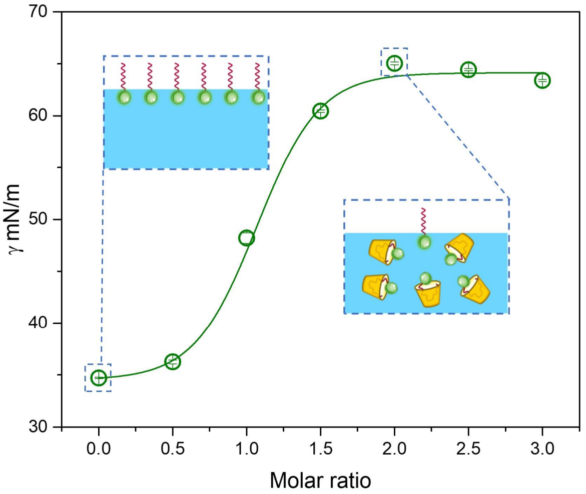

Supplementary Figure 12. Surface tension variations of SDBS with β-CD concentration. β-CD confines the hydrophobic chain of SDBS to its hydrophobic cavity, shielding the surface activity of SDBS. Error bars represent standard deviations (n=3).

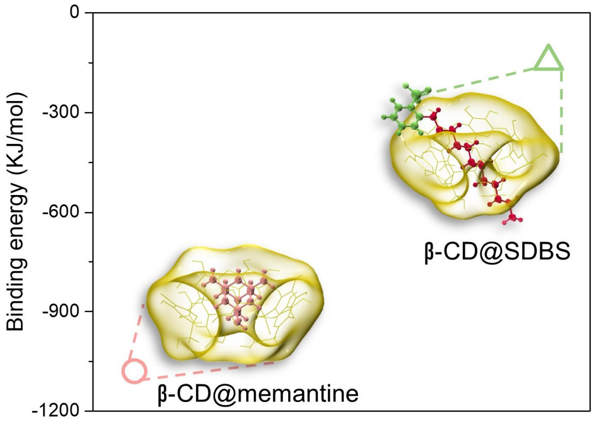

Supplementary Figure 13. Plots of the binding energy between the β-CD and guest molecules. The β-CD@memantine complexes have better thermodynamic stability.

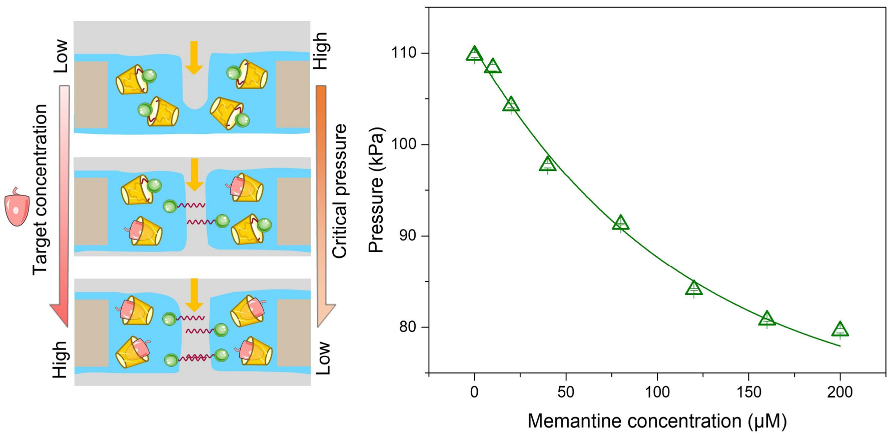

Supplementary Figure 14. Critical pressure variance with memantine concentration (0-200 μM). Memantine can displace the SDBS in the cavity of β-CD, resulting in the decrease of the critical pressure of the system. Error bars represent standard deviations (n=3).

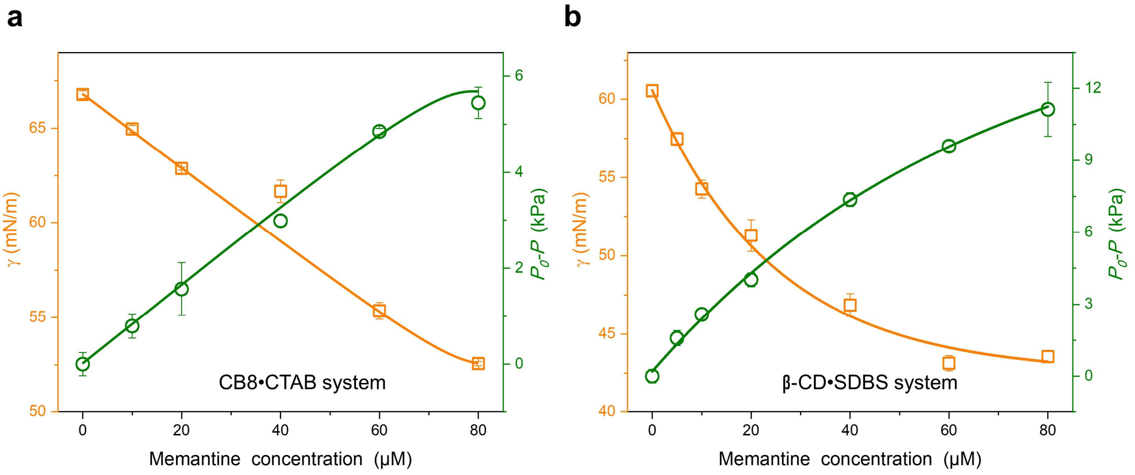

Supplementary Figure 15. Detection of memantine in urine from 0−80 μM with the (**a**) CB8•CTAB system and (**b**) β-CD•SDBS system. Error bars represent standard deviations (n=3).
